# HDAC1-mediated CDK1 decrotonylation inhibits colorectal cancer proliferation by regulating cell cycle and apoptosis

**DOI:** 10.3389/fonc.2026.1759160

**Published:** 2026-06-16

**Authors:** Dongling Li, Qinrui Cai, Ling Lin, Li Li, Yao Chen, Tianlin Feng, Xiaoya Zhou, Jia Xie, Xiaohong Fu, Chuanwei Li, Jun Xiao, Fan Yang

**Affiliations:** 1 Central laboratory of Chongqing Emergency Medical Center, Chongqing University Central Hospital; School of Medicine, Chongqing University, Chongqing, China; 2 College of Pharmacy and Bioengineering, Chongqing University of Technology, Chongqing, China; 3 Center Laboratory, The Affiliated Yongchuan Hospital of Chongqing Medical University, Chongqing, China; 4 Department of Cardiology, Chongqing Medical Emergency Center, Chongqing University Central Hospital, School of Medicine, Chongqing University, Chongqing, China

**Keywords:** Cdk1, CRC, crotonylation, epigenetics, PTMs

## Abstract

**Background:**

Colorectal cancer (CRC) represents the third most prevalent malignancy globally and ranks as the second leading contributor to cancer-related mortality. Lysine crotonylation, a novel post-translational modification, regulates chromatin dynamics through its dual substrate specificity targeting both histones and non-histone proteins.

**Methods:**

We quantitatively compared crotonylation levels between colorectal tumor specimens and paired adjacent normal tissues. The function and mechanism of specific crotonylation sites in hypercrotonylated proteins were further explored both *in vitro* and *in vivo* by introducing mutations that mimic decrotonylation at the crotonylation sites of CDK1.

**Results:**

Quantitative analysis revealed a significant elevation of global crotonylation in colorectal tumor specimens compared to adjacent normal tissues. Through LC-MS/MS analysis, we identified the CDK1 9th lysine crotonylated, and that decrotonylated of CDK1-K9 inhibited colorectal tumor proliferation and migration *in vitro* and *in vivo* by arresting the cell cycle at the G2/M phase and inducing apoptosis. Mechanistically, histone acetyltransferase hMOF (KAT8) and histone deacetylase HDAC1 co-mediated CDK1 K9 crotonylation, and the decrotonylated mutation was observed to decrease the interaction of CDK1 with cyclin and kinase activity. Moreover, CDK1 K9 decrotonylation and CDK1 inhibitor RO-3306 exert synergistic effects.

**Conclusion:**

We revealed the role of non-histone protein crotonylation in regulating the proliferation and migration of colorectal tumor. Crotonylation inhibited cell proliferation through the crotonylated CDK1 K9 by arresting the cell cycle and inducing apoptosis, and exerts a synergistic effect with the RO-3306 inhibitor.

## Graphical Abstract

**Figure:**
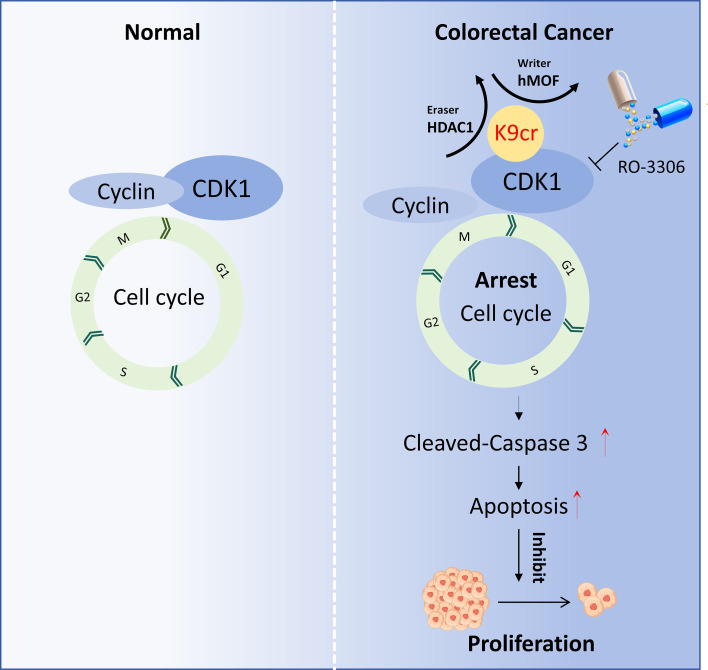
CDK1 decrotonylation inhibits proliferation in colorectal cancer and synergizes with inhibitor RO-3306. In colorectal cancer cells, CDK1 K9 crotonylation is regulated by the crotonyltransferase hMOF and the decrotonylase HDAC1. CDK1 K9 decrotonylation inhibited CRC cell proliferation through cell cycle arrest and induction of apoptosis. In addition, CDK1 K9 decrotonylation has a significant synergistic effect with the RO-3306 inhibitor.

## 1 Introduction

Colorectal cancer (CRC) is one of the most commonly diagnosed malignancies worldwide, with an increasing mortality and incidence (1). Epidemiological projections estimate a 60% surge in CRC disease burden by 2030 (2). It is therefore of paramount importance to achieve a comprehensive understanding of CRC proliferation, as it would serve to catalyze the development of new treatment approaches for patients (2).

The evolving understanding of protein regulation has highlighted the critical role of post-translational modifications (PTMs) in cellular pathophysiology. Previous studies have reported many novel modifications, such as methylation (3), formylation, acetylation, butyrylation, and lactylation (4). Many studies have investigated CRC of proliferation and metastasis at the genome level, but studies of rare PTMs in CRC is remarkably limited (5). Unlike the relatively stable genome and transcriptome, PTMs exhibit heightened complexity and diversity due to their reversible nature and regulation by various internal and external factors (6). Therefore, investigating the PTMs in CRC could provide a new insight into cancer research.

Lysine crotonylation (Kcr) shares structural homology with acetylation but exhibits distinct biochemical properties due to its extended aliphatic chain and conjugated π-system. This dynamic modification is orchestrated by three regulatory components: crotonyltransferases (writers), decrotonylases (erasers), and specific recognition modules (readers) (7). Many studies have identified that proteins crotonylation has a positive effect on cancer treatment (8). It has been reported that crotonylation was greatly elevated in CRC and other cancers (9). And current studies on Kcr in CRC are still insufficient, more studies and researches should be deeply put into this area.

CDK1 as one of the most major cell cycle checkpoint proteins, is involved of plenty fundamental cellular functions for cell proliferative growth (10–12). Previous studies have documented the existing modifications on CDK1, and their functions (13), and the expression of CDK1 is closely related to the survival of tumors (14, 15). However, the study of crotonylation on CDK1 remains elusive. In this study, we investigated the effects of crotonylation on CRC proliferation. Our analysis revealed that decrotonylation of CDK1 could impair CRC growth and metastasis, promote apoptosis and arrest the cell cycle. In addition, decrotonylation of CDK1 can increase the sensitivity of cells to CDK1 inhibitors and enhance drug efficacy. In conclusion, our study revealed a novel and essential feature of crotonylation in CRC proliferation.

## 2 Materials and methods

### 2.1 Patients and tissues

Human CRC tissues and the corresponding adjacent non-tumor tissues were obtained from the Affiliated Central Hospital of Chongqing University, Chongqing Emergency Medical Center, Chongqing, China, and the Ethics Committee from Chongqing Emergency Medical Center approved all aspects of this study (2024 Lunshen No. (52)). All participants provided written informed consent, and the study was approved by the local ethics committees. The studies were conducted in accordance with the Declaration of Helsinki and the International Ethical Guidelines for Biomedical Research Involving Human Subjects (CIOMS).

### 2.2 Cell lines and culture

MC38, CT26 and NIH3T3 cells were purchased from Cell Resource Center, Peking Union Medical College (PCRC), MC38 and CT26 were cultured in RPMI1640 supplemented with 10% fetal bovine serum and 1% penicillin/streptomycin, while the NIH3T3 was cultured in DMEM supplemented with 10% fetal bovine serum and 1% penicillin/streptomycin. All cell lines were authenticated by short tandem repeat (STR) profiling and tested as mycoplasma-free.

### 2.3 Establishment of stable-expressing cell lines

The overexpressed CDK1 wide type, mimic decrotonylation CDK1 K9R and NC were synthesized by Tsingke, China, and separately inserted into the vector Flag-tagged TK-PCDH-copGFP-T2A-Puro, and the resulting plasmids were labelled Flag-CDK1-WT, Flag-CDK1-K9R and NC. Subsequently, the packaged lentivirus, comprising the aforementioned three plasmids, was used to infect MC38 cells at a multiplicity of infection (MOI) of 10, followed by screening with puromycin (Solarbio Life Sciences, Beijing, China). The resulting MC38 cells with stable overexpression were named as the CDK1 WT and CDK1 K9R cell lines, and the control MC38 cells with stable expression of NC were named as the NC cell line.

### 2.4 Animal experiments

All animal care and handling procedures were performed in accordance with the National Institutes of Health Guide for the Care and Use of Laboratory Animals, and were approved by the Animal Ethics Committee of Chongqing University of Technology (Chongqing, China) (No. 2024048). Athymic male BALB/c nude mice (5 weeks old) were purchased from Huachuang Sino (Taizhou, China) and maintained under specific pathogen-free conditions. The sample size was chosen based on the literature and our previous experience, and for each experiment. Prior to carrying out the experiment, mice were randomly assigned to different groups. For subcutaneous injection studies, 1 × 106 cells were resuspended in 100 µl PBS and injected into the same place of the mice. Tumor growth was measured several weeks (2–4 weeks) following injection. The mice were euthanized by cervical dislocation to collect the tumors. Only healthy mice, free from infectious diseases and parasites, were selected, and mice with health issues, abnormal weight changes, or unexpected tumor growth were excluded. Blinding strategy when assessing the outcome was used whenever possible.

### 2.5 Cell viability assay

A total of 2000 cells were seeded into 96-well plates and cultured for 5 days. Subsequently, 10 μl of the CCK-8 solution was added to each well on a daily basis. Following a 60-minute incubation period at 37 °C, the optical density (OD) values at 450 nm were determined with a microplate reader (Tecan, Austria) and normalized against that of the corresponding control.

### 2.6 Colony formation assay

A total of 1000 cells were plated into six-well plates and cultured for approximately 12 days. Cell colonies were fixed with 4% formaldehyde (P0099, Beyotime) and stained with 0.1% Crystal violet (C8470, Solarbio) for 15 min, and the result of colonies was photographed and counted manually.

### 2.7 EdU assay

Cells are seeded into 24-well plates and grow for 24h. After reaching the desired confluence, EdU solution is added to the culture medium, and the cells are incubated for a period of 2–4 hours. Following incubation, the cells are fixed with 4% paraformaldehyde for 15 minutes and then permeabilized using 0.5% Triton X-100 for 15–20 minutes. Next, the reaction mix is added to label the incorporated EdU. The cells are then incubated for 30 minutes at room temperature. Finally, the images of the labeled cells were then observed and captured using microscopy.

### 2.8 Transwell assay

Cells are initially detached using trypsin-EDTA and resuspended in serum-free culture medium. Then the cell suspension (1 × 10^6^ cells/mL) is then added to the upper chamber of the Transwell and incubated for 10 minutes at 37 °C and 5% CO_2_ to allow the cells to settle. RPMI1640 supplemented with 10% fetal bovine serum and 1% penicillin/streptomycin is carefully added to the lower chamber. Culturing for 48 hours. After incubation, the migrated cells on the lower surface of the membrane were fixed with 4% formaldehyde (P0099, Beyotime) and stained with 0.1% Crystal violet (C8470, Solarbio) for 15 min, then counted under a microscope.

### 2.9 Western blot

The harvested cells were washed with the 1X iced-cold PBS and lysed in RIPA cell lysis buffer for 5 minutes, then the mixed buffer was subjected to ultrasonication for 10 minutes, after which it was centrifuged at 14,000 × g at 4 °C for 15 minutes. The protein lysates were separated on SDS-PAGE gels and transferred onto a 0.22 μm PVDF membrane for 2 hours. Skim milk powder was used for blocking for 2 hours. The membrane was then subjected to overnight incubation at 4 °C with antibodies. The next day, after three times wash in TBST, the membrane was incubated with the suitable secondary antibody for 1 hour at room temperature. Finally, the membrane was exposed by the VILBER image-forming system.

### 2.10 Antibodies and reagents

The following antibodies were used: anti-CDK1(ABclonal, Cat.No: A11420, 1:1000), anti-Beta Actin (HUABIO, Cat.No: EM21002, 1:10000), anti- lysine crotonylation (PTMa, Cat.No: PTM-501, 1:1000), anti-Flag (Abmart, Cat.No: M20003L, 1:1000), anti-HDAC1 (ABclonal, Cat.No: A0238, 1:1000), anti-P300 (Cell signaling technology, Cat.No: E8S2V, 1:1000), anti-CBP (Cell signaling technology, Cat.No: D6C5, 1:1000), anti-hMOF (HUABIO, Cat.No: ET7108-23, 1:1000), anti-GFP (Beyotime, Cat.No: AF1483, 1:1000), anti-P27 (Proteintech, Cat.No: 25614-1-AP, 1:1000), anti-p27 phospho Thr187 (Immunoway, Cat.No: YP0283, 1:1000), cyclinD1(Proteintech, Cat.No: 60186-1-Ig), CyclinE1 (ABclonal, Cat.No: A22461, 1:1000), CyclinB1 (Cell signaling technology, Cat.No: 4138S, 1:1000), Bcl2 (ABclonal, Cat.No: A19693, 1:1000), Bax (ABclonal, Cat.No: A20227, 1:1000), Cleaved Caspased3 (Affinity, Cat.No: AF7022, 1:1000), Ki67 (ABclonal, Cat.No: A20018, 1:200), anti-CDK1 K9 crotonylation (PTMa), Acetylated-Lysine Antibody (Cell signaling technology, Cat.No: 9441, 1:1000).

### 2.11 Co-immunoprecipitation

Cells were washed three times with 1 × PBS subsequently lysed on ice in lysis buffer (P0013, Beyotime). Co-IP assays were conducted by incubating anti-Flag magnetic beads (20565ES76, Yeasen Biotechnology) at 4 °C for one night. Then the beads were washed four times with ice-cold lysis buffer, and the proteins were denatured in 2×loading buffer by heating at 98 °C for 10 minutes. Finally, the eluted proteins were analyzed via western blotting.

### 2.12 Immunofluorescence

Following standard experimental protocols, the cells were initially fixed with Paraformaldehyde (4%), After thorough washing with PBS, block nonspecific binding by incubating the samples with a blocking buffer containing BSA for 60 minutes at room temperature. Subsequently incubated with primary antibodies at a dilution of 1:100. Subsequently, fluorescence dye-conjugated secondary antibodies were added, along with DAPI for nuclear staining. The immunofluorescence images of the labeled cells were then observed and captured using microscopy.

### 2.13 Measurement of Apoptosis and cell cycle

To analyze apoptosis using flow cytometry, cells are first cultured to the desired confluence and then detached using trypsin-EDTA. The cells are centrifuged to form a pellet and resuspended in a binding buffer. For staining, cells are incubated with Annexin V-FITC and propidium iodide (PI) according to the kit instructions. Typically, cells are incubated in the dark for 10–15 minutes at room temperature. After staining, the cells are analyzed using a flow cytometer.

To analyze the cell cycle using flow cytometry, cells are detached using trypsin-EDTA. After centrifugation to form a pellet, the cells are washed with PBS and fixed by adding 70% ethanol dropwise while vortexing gently. The fixed cells are stored at -20 °C for 2h. Before analysis, the cells are centrifuged to remove ethanol and resuspended in a staining solution containing propidium iodide (PI) and RNase A. The cells are then incubated in the dark at room temperature for 30 minutes. Finally, the cells are analyzed using a flow cytometer.

### 2.14 Statistical analysis

The data are presented as individual samples with the standard deviation (SD) indicated. The statistical significance of the differences between the WT and mutant groups was determined using Student’s two-tailed unpaired t-test or one-way ANOVA with GraphPad Prism software (GraphPad Software, Inc., La Jolla, CA, USA). Significant differences were observed when p values were <0.05.

## 3 Results

### 3.1 CDK1 is crotonylated at Lys9 in CRC

**Figure 1:**
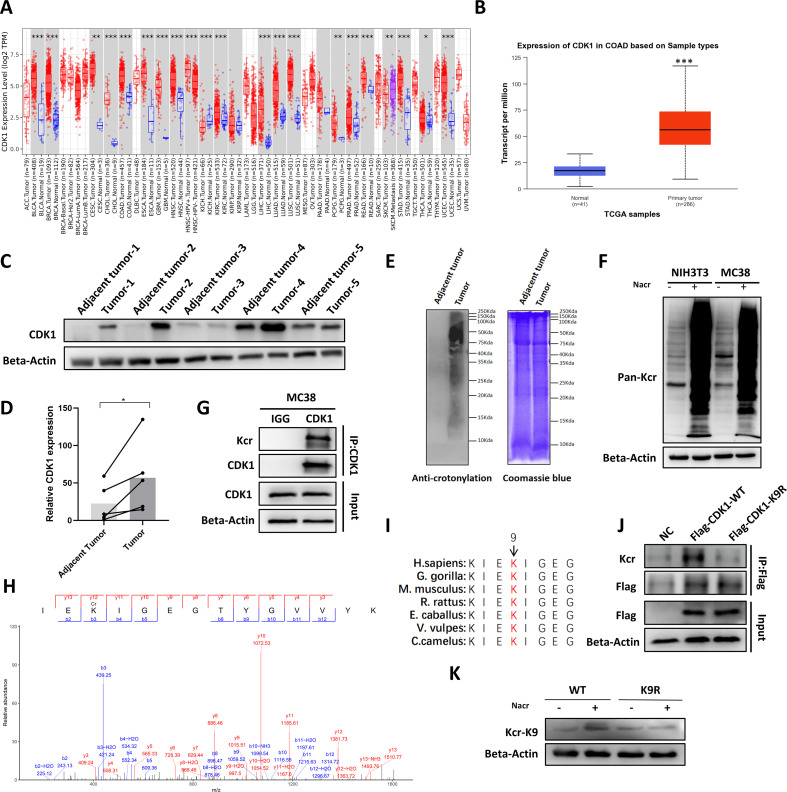
CDK1 is crotonylated at Lys9 in CRC. **(A)** Pan-cancer analysis of CDK1 expression via TIMER2.0. **(B)** The transcript levels of CDK1 in CRC were analyzed via CPTAC. **(C)** Western blotting showing that CDK1 is upregulated in tumor tissues. **(D)** Quantitative analysis of CDK1 protein expression in 5 paired colorectal cancer tissues and adjacent normal tissues by Western blot. **(E)** Western blotting revealed that tumor tissue with high crotonylation level. **(F)** Baseline crotonylation levels in MC38 versus NIH3T3 cell lines, with sodium crotonate (NaCr, 30 mM) treatment inducing significant crotonylation elevation in both lines. **(G)** Cell lysates were immunoprecipitated with CDK1 antibodies, followed by western blotting with an anti-pan-crotonylation (Kcr) antibody. **(H)** LC-MS/MS spectrum of CDK1 peptides containing crotonylated K9. **(I)** CDK1-K9 is evolutionarily conserved in seven species. K9 in CDK1 is highlighted in red. **(J)** Immunoprecipitated with the anti-Flag antibody and detected with anti-pan-Kcr antibody. **(K)**Western blot analysis of cells overexpressed Flag-tagged CDK1 WT and K9R, anti-CDK1-K9 crotonylation (Kcr-K9) antibody detected with or without Nacr treatment cells. ns p>0.05, *p<0.05, **p<0.01, ***p<0.001, ****p<0.0001.

Recent studies have found that CDK1 is closely related to proliferation and poor survival in many human cancers (16). To validate the CDK1 expression in CRC, we utilized the GEPIA tool to analyze its mRNA expression in pan-cancer based on TCGA data (17). Indicating that compared with the normal tissues CDK1 was highly expressed in several cancer types (**Figure 1A**), especially in CRC (**Figure 1B**). We also collected the tumor and adjacent tumor tissues from clinical CRC patients, and the western blotting result showed the same result that CDK1 was commonly highly expressed in tumor tissues of CRC patients (**Figures 1C, D**).

Studies have demonstrated that lysine crotonylation exerts beneficial effects in cancer treatment (16). To investigate the potential association between crotonylation and CRC, an analysis was conducted to compare the crotonylation levels in tumor tissues and adjacent tissues from CRC patients. As illustrated in **Figure 1E**, tumor tissues exhibited more hypercrotonylated proteins. Research indicates that sodium crotonate (NaCr) enhances Kcr modification by elevating intracellular crotonyl-CoA levels in cell cultures (17). We subsequently analyzed baseline crotonylation levels in two cell lines and their responses to NaCr treatment. NIH3T3 cells displayed lower basal crotonylation than MC38 cells, with NaCr significantly increasing crotonylation in both lines (**Figure 1F**; **Supplementary Figure 1A**).

Utilizing immunoprecipitation (IP) to enrich CDK1 in MC38 cells, the presence of crotonylation of CDK1 was identified (**Figure 1G**). Moreover, Mass spectrometry identified Lys9 (K9) as the primary crotonylation site on CDK1 (**Figure 1H**). Analyzing CDK1 protein sequence alignments across different species, the study revealed that the amino acid residue K9 was highly evolutionarily conserved (**Figure 1I**). Compared with the CDK1 wide type, the CDK1 mutant, in which the lysine residue at position 9 has been replaced with arginine, the mutation substantially reduced CDK1 crotonylation compared to wild-type CDK1(**Figure 1J**; **Supplementary Figures 1B, H**). Antibody validation demonstrated specific recognition of CDK1-K9 crotonylation by dot blot (**Supplementary Figure 1C**) and immunoblot (**Figure 1K**).

### 3.2 CDK1-K9 decrotonylation inhibits CRC cell proliferation *in vivo* and vitro

**Figure 2:**
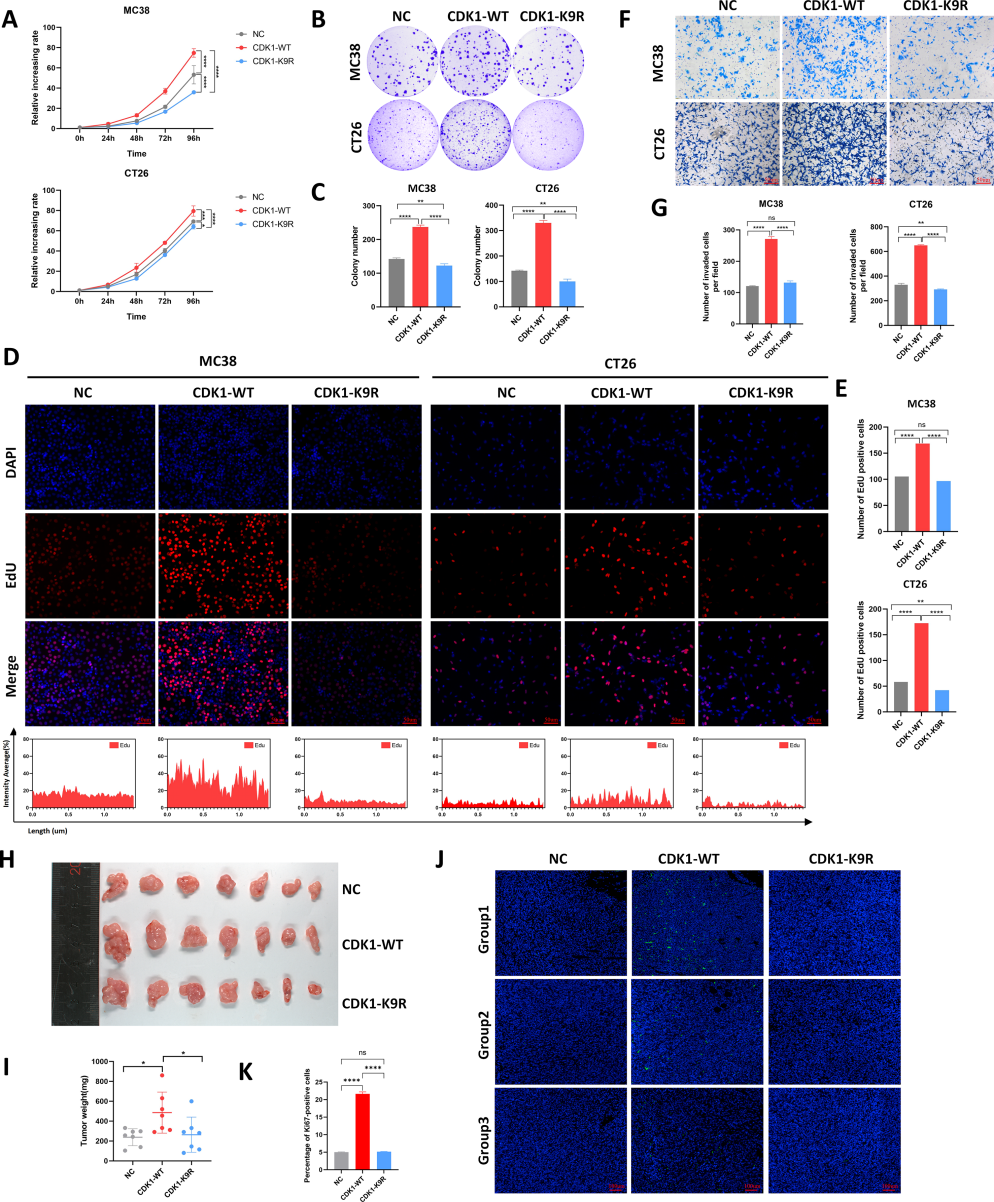
CDK1-K9R mutant inhibited CRC cell proliferation and migration *in vivo* and vitro. **(A)** CCK8 assay in MC38 and CT26 cells. **(B)** Representative images of colony formation assays (n=3) in MC38 and CT26 cells. **(C)** Quantification of the number of colonies formed. **(D)** EdU assay results for MC38 and CT26 cells, together with a statistical chart (magnification, 50um). **(E)** Quantification of the number of EdU positive cells. **(F)** Transwell in MC38 and CT26 cells (scale bar: 50um). **(G)** Quantification of the number of transwell cells. **(H)** Tumors 12 days post-injection of WT or K9R mutant MC38 cells. **(I)** Quantitative comparison of the number of tumor weights in each group. **(J)** Immunofluorescence image showing that CDK1-WT group tumor had high levels of Ki67 (magnification, 100um) Representative immunofluorescence images of Ki67 expression in xenograft tumors (scale bar: 100 µm). **(K)** Quantitative analysis of Ki67 expression in different groups. The data are presented as the means ± SD. ns p>0.05, *p<0.05, **p<0.01, ****p<0.0001.

To elucidate the functional role of CDK1-K9 crotonylation, we generated stable cell lines overexpressing wild-type Flag CDK1 WT or the Flag CDK1 K9R mutant in the colorectal cancer cell lines MC38 and CT26, and the western blot verified the stable overexpression of CDK1 WT and CK1 K9R mutant MC38 cells (**Supplementary Figure 1C**). We first carried out the cell counting kit (CCK)-8 assay and found that compared with the wide type, the CDK1 mutant significantly reduced the viability of two cells, which greatly inhibit the colorectal cancer cell proliferation (**Figure 2A**). Similarly, the number of clones formed by mutant cells dropped even lower to the level of control cells (**Figures 2B, C**). EdU can be taken up by proliferating cells and bind with DNA, thereby labeling the cells undergoing DNA replication. We used the EdU (5-ethynyl-2’-deoxyuridine) labeling method to detect the proliferative activity of CRC-overexpressing cell lines. It was observed that in the CDK1 WT group, the EdU-positive cells had a stronger red fluorescence signal, and accounted for approximately 80% of the total cells. In contrast, the number and fluorescence intensity of EdU-positive cells were significantly reduced in the CDK1 K9R group, accounting for only approximately 20% of the total number of cells (**Figures 2D, E**). We also observed that the CDK1 K9R mutant significantly suppressed the migration of CRC cells compared with CDK1 WT and control (**Figures 2F, G**). These results imply that K9 decrotonylation could inhibit cell growth and migration.

To validate our findings *in vivo*, we established subcutaneous mouse models by injecting overexpressed WT CDK1, CDK1 K9R MC38 cells into BALB/c nude mice (n=7 per group). The mice were sacrificed 12 days later, and the subcutaneous tumors were successfully created. The results showed that the weights of the mice were not statistically significantly different, but the tumor volumes and weights of the mice in the CDK1 K9R group were significantly lower than those in the CDK1 WT group (**Figures 2H, I**; **Supplementary Figures 1D, E**). We performed Immunofluorescence staining for Ki67 in paraffin-embedded tumor tissue from the mouse samples. And the results showed that the fluorescence intensity and positive cell count were significantly lower in the CDK1-K9R groups than in the WT group (**Figure 2J**). These results suggest that the crotonylation of CDK1 plays an important role in the regulation of CRC progression.

### 3.3 CDK1 K9 decrotonylation reduces CDK1 kinase activity and induces apoptosis

**Figure 3:**
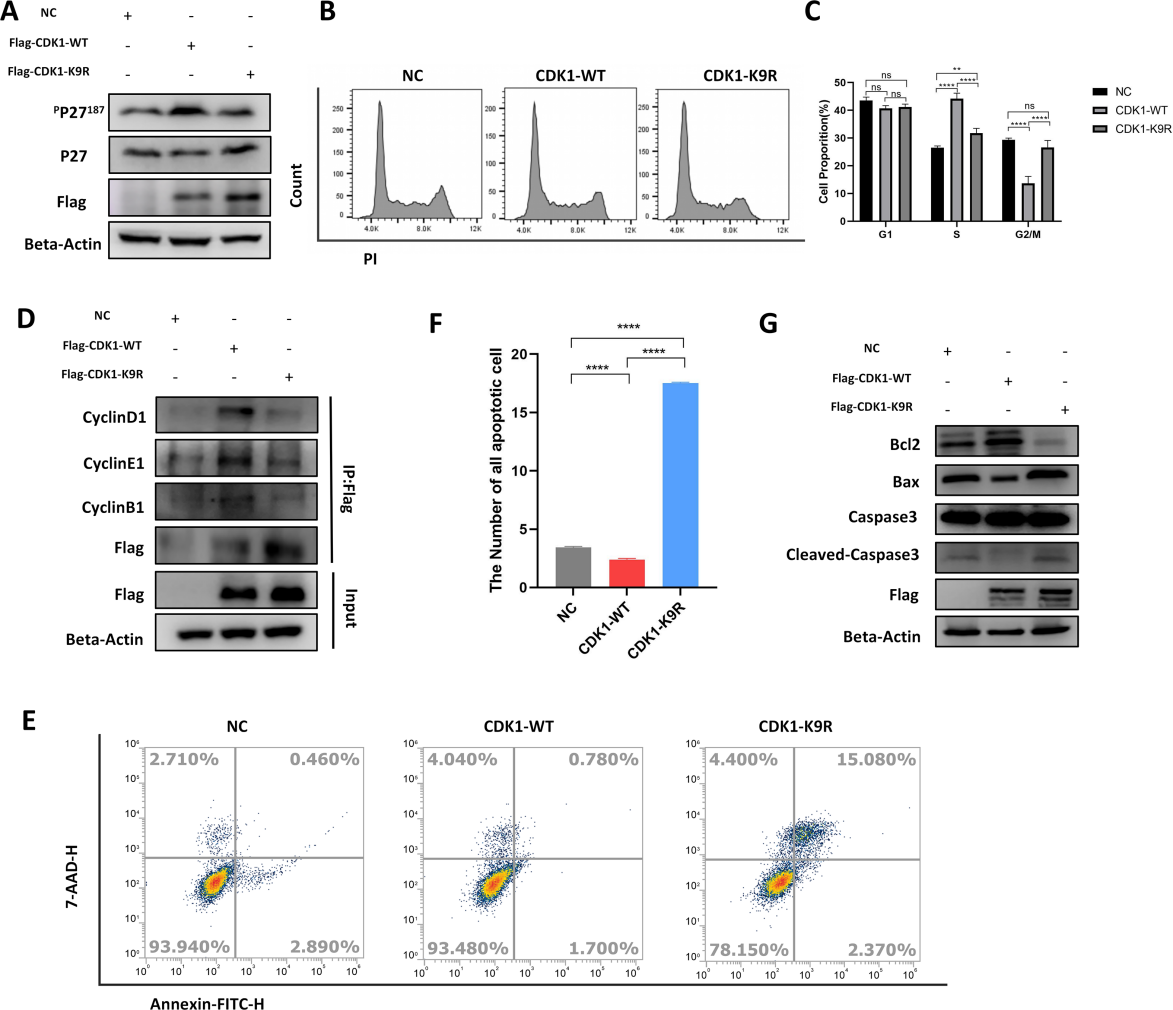
CDK1 K9R reduced CDK1 kinase activity and induced apoptosis. **(A)** CDK1 kinase activity assay. **(B)** Flow cytometry was used to detect the cell cycle in MC38 cell. **(C)** Quantification of the results of the flow cytometry cell cycle assay (n = 3). **(D)** CDK1 K9R overexpression decreased the interaction with cyclin proteins in MC38 cell. **(E)** Flow cytometry was used to detect the cell apoptosis. **(F)** Quantification of the results of the flow cytometry apoptosis assay (n = 3). **(G)** Western blot shows that CDK1 K9R promotes the cell apoptosis, whereas CDK1 WT rescues the cell apoptosis. The data are presented as the means ± SD. ns p>0.05, **p<0.01, ****p<0.0001.

A previous study has revealed that the overexpression of CDK1 increased activity of CDK1 which in turn phosphorylates the Thr187 site of P27, leading to a reduction in p-P27 (18). It was evident that the K9R mutation resulted in a significant reduction in CDK1 activity when compared to the WT (**Figure 3A**). CDK1 is a crucial protein in the cell cycle. Flow cytometric analysis of the cell cycle distribution revealed that the proportion of cells in the G2/M phase in the K9R mutant was significantly greater than that in the WT, and the proportion in the S phase was lower than that in the WT. These findings suggest that K9R inhibited cell growth by arresting the cell cycle at the G2/M phase (**Figures 3B, C**). We subsequently investigated the binding capacity of these cell cycle proteins, and the findings indicated a marked decrease in the interaction of the mutant CDK1 and cyclinB1, cyclinD1 and cyclinE1 (**Figure 3D**). Additionally, after the cells were stained with 7-AAD and Annexin-V-FITC, the flow cytometry results showed that K9R observably increased the proportion of apoptotic cells (**Figures 3E, F**). Western blot results showed that in the CDK1 K9R groups, the expression level of the antiapoptotic protein BCL-2 was markedly reduced, whereas the expression level of the proapoptotic protein Bax was significantly elevated. These findings were coincided with the expression of total caspase3 and cleaved-caspase3 (**Figure 3G**).

### 3.4 hMOF and HDAC1 co-regulate the crotonylation dynamics of CDK1

**Figure 4:**
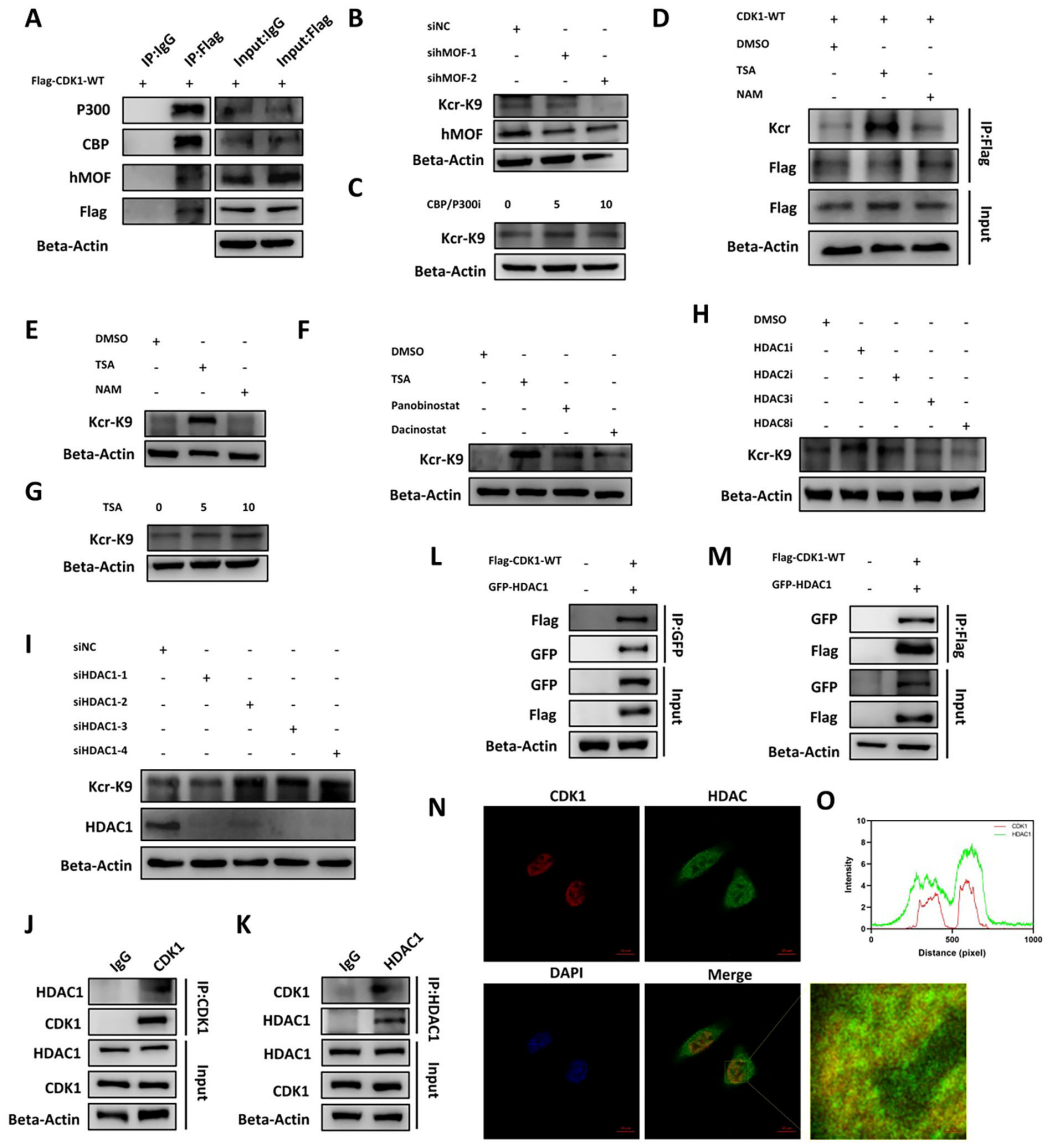
hMOF and HDAC1 co-regulate the dynamic crotonylation of CDK1. **(A)** Cell lysates were immunoprecipitated with Flag antibodies, followed by western blotting (WB) with CBP, P300, hMOF, and FLAG antibodies. **(B)** Knockdown of hMOF impairs CDK1 crotonylation in MC38 cell. **(C)** Functional analysis of the effects of CBP/P300 inhibitor treatment on the decrotonylation of CDK1. **(D)** Functional analysis of the effects of TSA and NAM treatment on the decrotonylation of CDK1. **(E)** Anti-CDK1-K9 crotonylation detected the effects of TSA and NAM treatment on the decrotonylation of CDK1. **(F)** Anti-CDK1-K9 crotonylation detected the effects of TSA, Panobinosta and Dacinostat treatment on the decrotonylation of CDK1. **(G)** Anti-CDK1-K9 crotonylation detected different concentrations of TSA on the decrotonylation of CDK1. **(H)** Western blot was performed to identify the key regulating decrotonylase for CDK1 with anti-CDK1-k9 crotonylation antibodies, HDAC1i (Valproic acid), HDAC2i (CAY10682), HDAC3i (RGFP966), HDAC8i (PCI-34051). **(I)** Knockdown of HDAC1 impairs CDK1-K9 crotonylation in MC38 cell. **(J, K)** CDK1 interacts with HDAC1 *in vivo*. **(L, M)** CDK1 interacts with HDAC1 *in vitro*. **(N, O)** Immunofluorescence image showing colocalization of HDAC1 and CDK1, and qualitative analysis of colocalization fluorescence (magnification, 10um and 1um).

We detected that CDK1 WT overexpressed interacting crotonyltransferases, CBP, P300 and hMOF in MC38 cells. The results showed that CBP, P300 and MOF all exhibited the capacity to interact with CDK1 (**Figure 4A**). We first transfected the sihMOF into MC38 cell demonstrated that with the decreasing level of MOF, the CDK1-K9 crotonylation also decreased (**Figure 4B**; **Supplementary Figure 1I**). Furthermore, following the CBP/P300 inhibitor treatment in MC38, it was observed that as the concentration of the inhibitor increased, the level of CDK1-K9 crotonylation remained unaltered (**Figure 4C**). All the results indicated that MOF has the capacity to crotonylate CDK1, whereas others exerted no impact on CDK1 crotonylation. Furthermore, we treated the NAM (SIRT inhibitors) and TSA (HDAC inhibitors) into over-expressed CDK1 WT MC38 cells, and IP enriched FLAG-tagged CDK1 to detect the Kcr. The result demonstrated that the TSA treatment group exhibited a significantly elevated Kcr level in comparison to the other groups (**Figure 4D**). We also added the inhibitors into the MC38 cells, the CDK1 K9 crotonylation antibody detected revealed a similar outcome (**Figure 4E**). Two additional HDAC inhibitors, Panbinostat and Dacinostat, were employed and the results indicated that HDACs was the primary decrotonylases for CDK1 crotonylation (**Figure 4F**). Furthermore, we observed a dose-dependent effect of TSA on CDK1 (**Figure 4G**). The main decrotonylases in HDACs were HDAC1, HDAC2, HADC3 and HDAC8 (19). Consequently, we proceeded to introduce the inhibitors of the aforementioned enzymes separately to the MC38 cells.

The results demonstrated that following the addition of the HDAC1 inhibitor, there was a notable increase in the expression of CDK1-K9 crotonylation (**Figure 4H**; **Supplementary Figure 1J**). This phenomenon was further confirmed by the interference of endogenous HDAC1 (**Figure 4I**; **Supplementary Figure 1K**). Subsequently, we conducted further investigations into the interaction between CDK and HDAC. We separately examined the interaction of endogenous and overexpressed Flag-tagged CDK1 and GFP-tagged HDAC1, and the results indicated that there is a clear interaction between them, regardless of whether it is endogenous or exogenous (**Figures 4J–L**). The results of immunofluorescence also revealed that there was significant co-localization between endogenous CDK1 and HDAC1 (**Figure 4N**). Qualitative analysis of the results further demonstrated this co-localization (**Figure 4O**). These findings indicated that CDK1 crotonylation may be regulated by HDAC1 and hMOF, and they do not affect the acetylation levels of CDK1 (**Supplementary Figures 1F, G**).

### 3.5 Synergistic tumor suppression by CDK1 K9R and RO-3306

**Figure 5:**
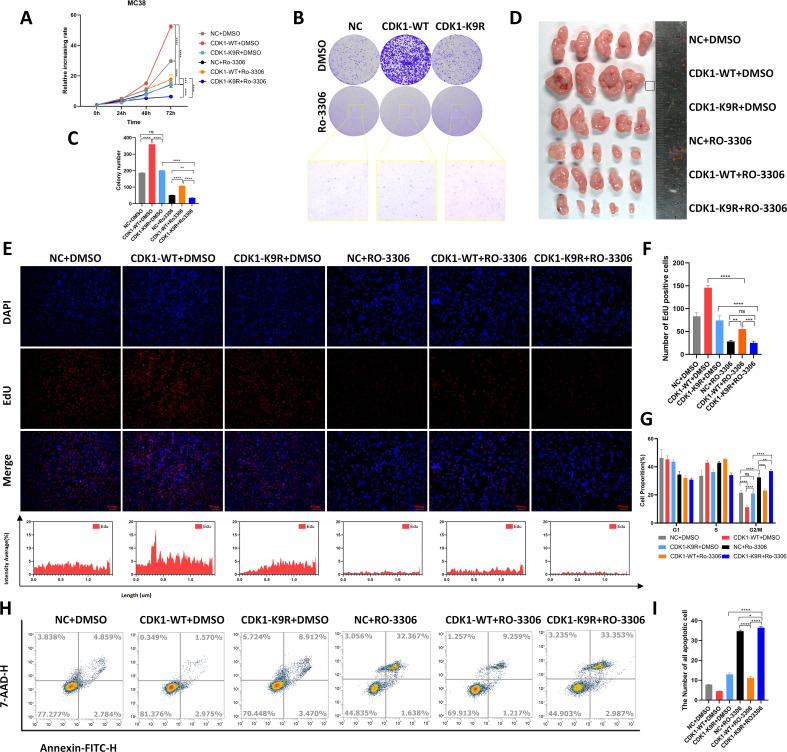
Synergistic tumor suppression by CDK1 K9R and RO-3306. **(A)** CCK8 assay in MC38 cell. **(B)** Colony formation (n=3) assay in MC38 cells and statistic analysis. **(C)** Quantification of the number of colonies formed. **(D)** Tumors 31 days post-injection of WT or K9R mutant MC38 cells and administer the CDK1 inhibitor Ro-3306 via intraperitoneal injection in mice once a week. **(E)** EdU assay in MC38 cell, together with its statistical chart (magnification, 50um). **(F)** Quantification of the number of EdU positive cells. **(G)** Flow cytometry was used to detect the cell cycle in MC38 cell, and the results of the flow cytometry cell cycle assay were quantified (n = 3). **(H)** Flow cytometry was used to detect the cell apoptosis. **(I)** Quantification of the results of the flow cytometry apoptosis assay (n=3). The data are presented as the means ± SD. ns p>0.05, *p<0.05, **p<0.01, ***p<0.001, ****p<0.0001.

RO-3306 is a CDK1 inhibitor that specifically targets and inhibits the catalytic activity of CDK1, thereby inducing cell cycle arrest in the G2/M phase and preventing cells from progressing through mitosis (20). Owing to its distinctive inhibitory effects, studies have shown its potential for anti-cancer applications (21, 22). After RO-3306 treatment, the CCK8 and colony formation results revealed that CDK1 WT group exhibited a reduction in cell growth and the outcome was equivalent to CDK1 K9R. It is also noteworthy that the CDK1-K9R group treated with RO-3306 exhibited significantly enhanced anti-tumor efficacy in comparison to the untreated CDK1 K9R (**Figures 5A–C**). We first injected overexpressed NC, WT CDK1, and CDK1 K9R MC38 cells into BALB/c nude mice (n=5 per group) to establish subcutaneous mice models. After 7 days, we administered the CDK1 inhibitor Ro-3306 via intraperitoneal injection in mice once a week, and the mice were sacrificed 24 days later. The results were the same as those of the CCK8 and colony formation results (**Figure 5D**; **Supplementary Figure 1L**). Furthermore, the EdU results corroborated this conclusion (**Figures 5E, F**). Flow cytometric analysis of the cell cycle revealed that the majority of cells in the CDK1 K9R group treated with RO-3306 were arrested at the G2/M phase (**Figure 5G**). The flow cytometry results demonstrated that the proportion of apoptotic cells was significantly elevated in the CDK1 K9R group following RO-3306 treatment (**Figures 5H, I**). All the results indicated that the CDK1 K9R mutation and the CDK1 inhibitor RO-3306 act in a synergistic manner to markedly suppress tumor progression.

## 4 Discussion

The discovery of a novel PTMs, lysine crotonylation, in 2011 marked a significant advancement in our understanding of protein PTMs (7). Numerous previous studies have performed comprehensive genomic analysis on non-histone crotonylation, and identified a close relationship between lysine crotonylation and a range of cellular functions (23, 24). Nevertheless, most studies have been limited to the analysis of proteins that can potentially undergo crotonylation, and the precise locations and functions of the protein modifications remain uncertain. In our work, we observed a high crotonylation level of tumor tissue, and identified the 9th lysine of CDK1 was crotonylated. Furthermore, this modification was observed to affect CRC growth by arresting cell cycle at G2/M phase and inducing apoptosis. This process is co-mediated by crotonyltransferase hMOF and decrotonylase HDAC1.

Crotonylation has also been detected in depression (25, 26), kidney disease (27), alzheimer’s disease (28), hypertrophic cardiomyopathy (29) and HIV (30), with studies identifying a close relationship with histone crotonylation. In non-small-cell lung cancer cells, the crotonylation of BEX2 at the K59 site has been identified as a critical mediator of mitophagy (31). Furthermore, crotonylation has been demonstrated to facilitate cell invasion through the crotonylated SEPT2-K74- P85α-AKT pathway in hepatocellular carcinoma (32). Decrotonylation of MTHFD1 resulted in increased resistance to ferroptosis and promoted pancreatic cancer (33). ENO1 has been identified as a crotonylated protein that regulates gene expression to promote CRC cell growth and migration (34). The activity and stability of CDK1 are intricately modulated by many PTMs that are pivotal to cell cycle regulation. Phosphorylation, notably at residues Y15, T14, and T161, critically governs CDK1’s interaction with Cyclin B1 and its subsequent kinase activation (35, 36). Ubiquitination at K48 exerts a significant influence on CDK1 stability, whereas SUMOylation serves to inhibit CDK1 activity, thereby contributing to cell cycle control (37, 38). Acetylation, by modulating CDK1’s association with Cyclin B1, also plays a role in regulating CDK1 function. Collectively, these PTMs orchestrate the precise regulation of CDK1 throughout the cell cycle (39). Against this backdrop, our study of crotonylation at the K9 site of CDK1 reveals a novel regulatory mechanism that is distinct from the established PTMs, thereby expanding the repertoire of known regulatory pathways for CDK1. Similarly, previous mass spectrometry studies have also detected crotonylation at the CDK1 K9 site, although it was not extensively investigated (23, 40). Compared with these studies, our research has also identified the specific modification sites and elucidated the underlying mechanisms. Moreover, we observed a significant synergistic effect between the CDK1 K9R mutant and the RO-3306 inhibitor. The combined use of CDK1 K9R and RO-3306 was more effective in inhibiting cancer cell growth than the use of either compound alone. Although the specific mechanisms were not extensively explored in our manuscript, we hypothesize that the CDK1 K9R mutant may inhibit kinase activity by altering the conformation of CDK1 or affecting its interactions with other proteins. Similarly, RO-3306 exerts its effect by inhibiting the functional domain of CDK1. The distinct functional regions targeted by these two agents may contribute to the altered functionality and the pronounced anticancer effects observed.

In this study, we have identified the precise crotonylation site of CDK1 and the mechanism involved. Nevertheless, it remains unclear whether the crotonylation in conjunction with others modifications to affect CRC proliferation, such as phosphorylation and acetylation. The balance between crotonylation and other modifications of metabolic enzymes that are co-regulated in CRC proliferation and metastasis remains to be elucidated. Moreover, although we investigated the impact of CDK1 crotonylation influence on inhibitor, we did not clarify the underlying mechanism. Furthermore, our investigation was limited to a single inhibitor, and we did not pursue further research on combinations with clinically relevant drugs. However, the precise mechanisms and functions underlying the regulation of the cell cycle and apoptosis by CDK1 crotonylation in CRC have not been fully experimentally validated.

Our study reveals that the CDK1-K9R mutant exhibits significantly reduced proliferation compared to wild-type CDK1 and the negative control (NC). This observation is consistent with our experimental findings that the K9R mutation inhibits CDK1’s kinase activity and impairs its ability to bind cyclins, which are essential for cell cycle progression (**Figures 3A, D**). The K9R mutation, intended to mimic the de-crotonylated state of CDK1, appears to have a pronounced effect on CDK1’s function. Specifically, the loss of crotonylation at lysine 9 may disrupt the structural integrity of CDK1, thereby affecting its catalytic activity and interactions with cyclins. These findings highlight the critical role of crotonylation in regulating CDK1’s function and suggest that this post-translational modification is essential for maintaining normal cell cycle control. Further investigation into the molecular mechanisms underlying the effects of the K9R mutation on CDK1’s function and its broader implications for cell cycle regulation is warranted. From a structural perspective, RO-3306 directly inhibits CDK1 activity by targeting the ATP-binding pocket within the kinase domain (41, 42). In contrast, the K9 residue resides in the flexible N-terminal tail, spatially separated from the active site (43). The K9R mutation indirectly reduces CDK1 function by altering local conformation and stability rather than blocking catalysis. Therefore, RO-3306 and K9R act through distinct structural regions and regulatory mechanisms, leading to synergistic suppression. Moreover, mutation at K9 does not disrupt the ATP-binding pocket, supporting that RO-3306 remains effective toward CDK1.

In conclusion, our study illuminated a previously unidentified function of CDK1 crotonylation in regulating the CRC cells proliferation and the synergistic action with inhibitor RO-3306. It may therefore be surmised that a more comprehensive understanding of the crotonylation process may open up new potential therapeutic targets for the prevention and treatment of CRC, also personalized medicines for patients may benefit from our research.

## Data Availability

The original contributions presented in the study are included in the article/**Supplementary Material**. Further inquiries can be directed to the corresponding authors.

## Supplementary material

Supplementary Figure 1 **(A)** WB analysis of total crotonylation of in MC38 cell lines after Nacr treatment. **(B)** Dot blotting assay with a site-specific antibody against CDK1-K9 crotonylation (K9Cr). **(C)** Western blot analysis of the over-expressed stable of CDK1-WT and CK1-K9R mutant MC38 cells. **(D)** The mice were weighed every 3 days and were sacrificed 12 days after the injection. **(E)** The mice tumor volumes were measured every 3 days and were sacrificed 12 days after the injection. **(F)** Functional analysis of the effects of knock down of hMOF on the acetylation of CDK1. **(G)** Functional analysis of the effects of knock down of HDAC1 on the acetylation of CDK1. **(H)** Immunoprecipitated with the anti-Flag antibody and detected with anti-pan-Kac, anti-pan-Klac, and anti-pan-Kcr antibody. **(I)** Statistical Analysis of hMOF Knockdown on CDK1 Crotonylation in MC38 Cells. **(J)** Statistical Analysis of HDAC Inhibitor Effects on CDK1-K9 Crotonylation in MC38 Cells. **(K)** Statistical Analysis of HDAC1 Knockdown on CDK1-K9 Crotonylation in MC38 Cells. **(L)** Quantitative comparison of the number of tumor weight in each group. The data are presented as the means ± SD. ns p>0.05, **p<0.01, ***p<0.001, ****p<0.0001.
